# Supplementary material for: The advantages of radial trajectories for vessel-selective dynamic angiography with arterial spin labeling
Source: MAGMA. 2019 Aug 17;32(6):643–53. doi: 10.1007/s10334-019-00771-1 (PMC6825642; doi:10.1007/s10334-019-00771-1)
Supplement: Supplementary file 1 — Supplementary file1 Online Resource 1: Supplementary figures showing example brain, noise and signal masks used for SNR calculations and inverted grayscale versions of Figures 3-6. (PDF 903 kb) [file 10334_2019_771_MOESM1_ESM.pdf]

Supplementary Figures

Article details:

Title: The advantages of radial trajectories for vessel-selective dynamic angiography with arterial spin labeling

Journal: *Magnetic Resonance Materials in Physics, Biology and Medicine*

Authors: Eleanor S. K. Berry[a], Peter Jezzard[a], and Thomas W. Okell[a,*]

Affiliations: [a]Wellcome Centre for Integrative Neuroimaging, FMRIB, Nuffield Department of Clinical Neurosciences, University of Oxford, Headley Way, Oxford, OX3 9DU, United Kingdom

*Email: tokell@fmrib.ox.ac.uk

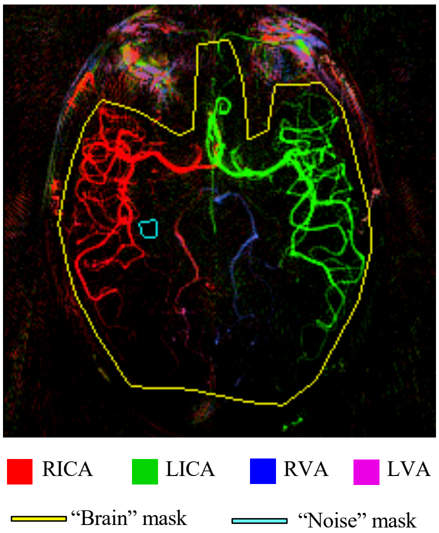

Supplementary Figure 1: Example masks used for SNR calculations overlaid on a 2D temporal mean data set. The brain mask (outlined in yellow) is used to exclude the scalp and eyes to avoid artifacts interfering with the SNR calculation. The small noise mask (outlined in cyan) is placed close to the arteries of interest near the centre of the brain, but avoiding any vessel signal.

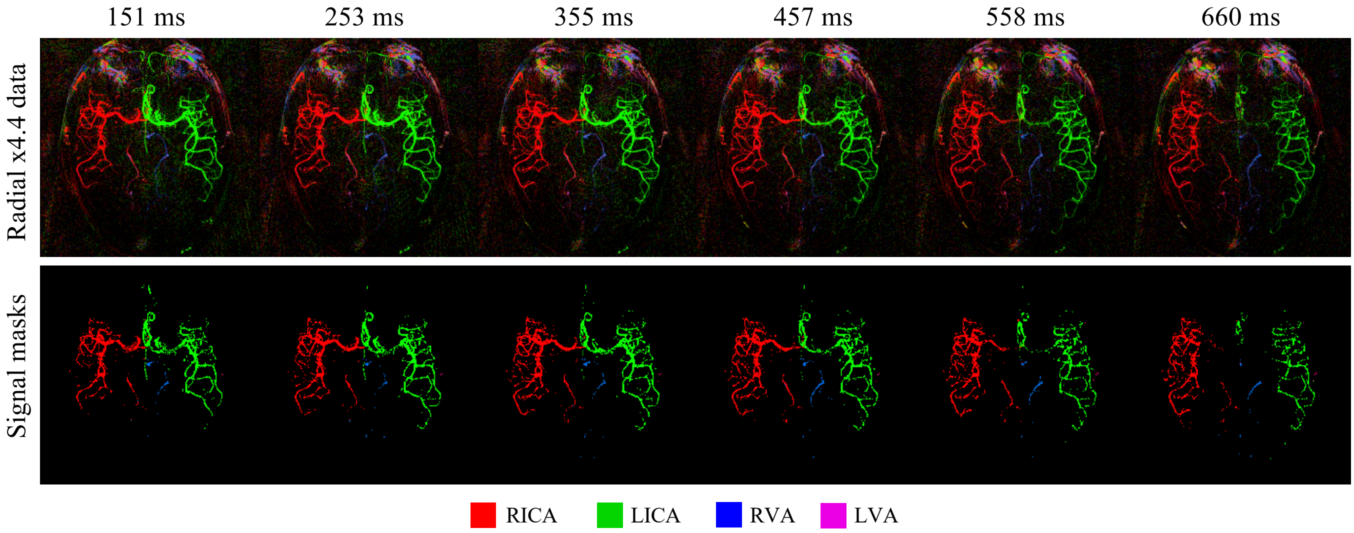

Supplementary Figure 2: Example frames from a 2D radial x4.4 undersampled data set (top) along with the signal masks used for the SNR calculation within each corresponding frame (bottom). Note that the signal masks have been color-coded in the same way as the data: only signal from the dominant arterial component in each of these voxels is used for the SNR calculation.

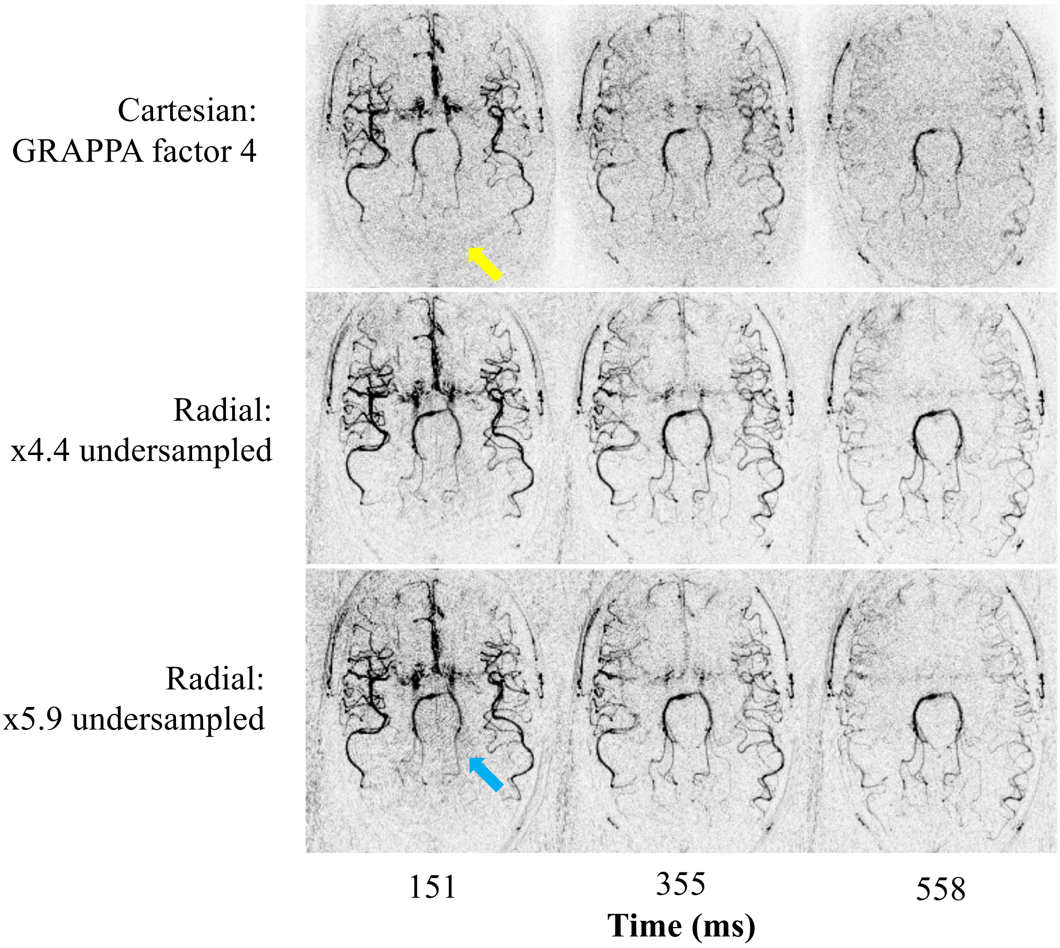

Supplementary Figure 3: Inverted grayscale version of Figure 3.

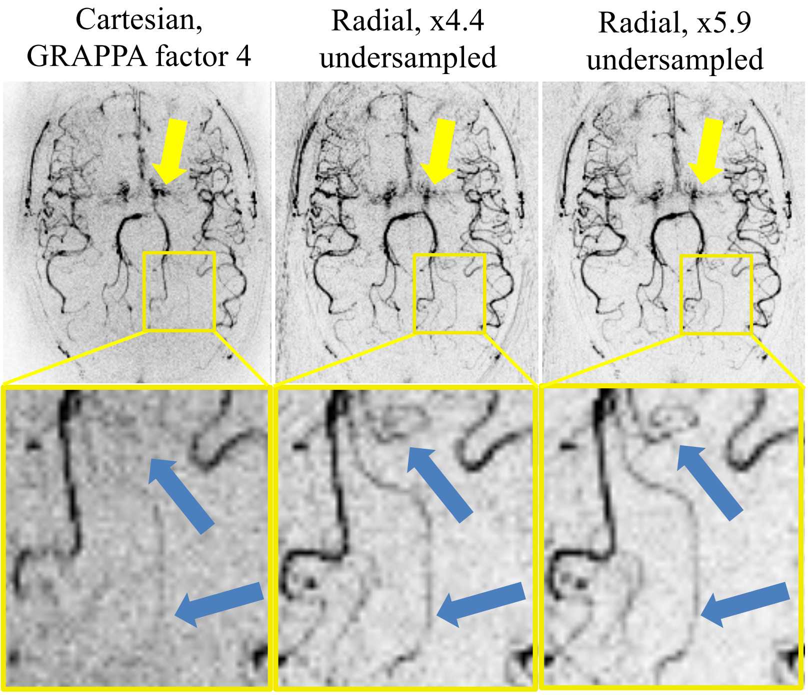

Supplementary Figure 4: Inverted grayscale version of Figure 4a.

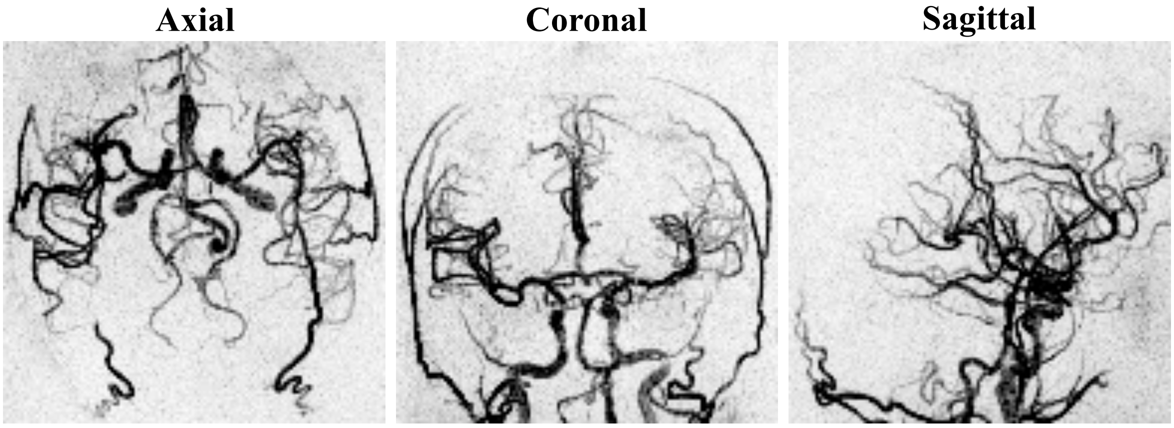

Supplementary Figure 5: Inverted grayscale version of Figure 5.

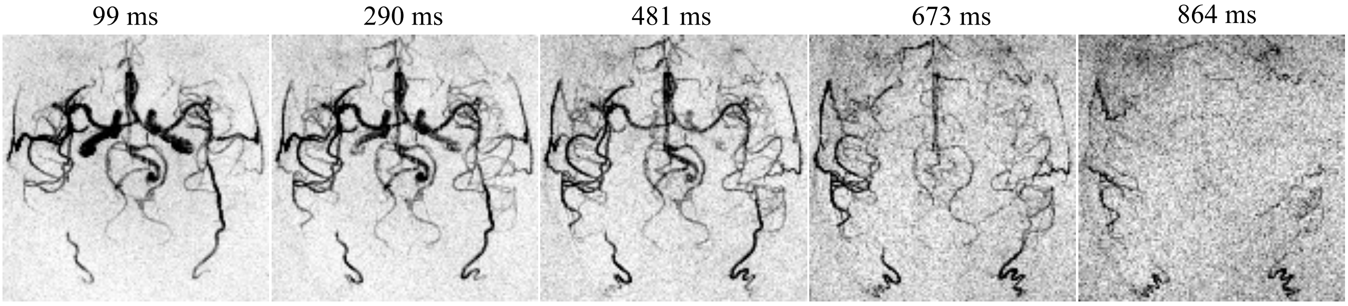

Supplementary Figure 6: Inverted grayscale version of Figure 6.
